# QTL Mapping Based on Different Genetic Systems for Essential Amino Acid Contents in Cottonseeds in Different Environments

**DOI:** 10.1371/journal.pone.0057531

**Published:** 2013-03-29

**Authors:** Haiying Liu, Alfred Quampah, Jinhong Chen, Jinrong Li, Zhuangrong Huang, Qiuling He, Shuijin Zhu, Chunhai Shi

**Affiliations:** Department of Agronomy, College of Agriculture and Biotechnology, Zhejiang University, Hangzhou, People's Republic of China; Nanjing Agricultural University, China

## Abstract

Cottonseeds are rich in various essential amino acids. However, the inheritance of them at molecular level are still not defined across various genetic systems. In the present study, using a newly developed mapping model that can analyze the embryo and maternal main effects as well as QTL × environment interaction effects on quantitative quality trait loci (QTLs) in cottonseeds, a study on QTL located in the tetraploid embryo and tetraploid maternal plant genomes for essential amino acid contents in cottonseeds under different environments was carried out, using the immortal F_2_ (IF_2_) populations from a set of 188 recombinant inbred lines derived from an intraspecific hybrid cross of two upland cotton germplasms HS46 and MARKCBUCAG8US-1-88 as experimental materials. The results showed a total of 35 QTLs associated with these quality traits in cottonseeds. Nineteen QTLs were subsequently mapped on chromosome 5, 6 and 8 in sub-A genome and chromosome 15, 18, 22 and 23 in sub-D genome. Eighteen QTLs were also found having QTL × environment (QE) interaction effects. The genetic main effects from QTLs located on chromosomes in the embryo and maternal plant genomes and their QE effects in different environments were all important for these essential amino acids in cottonseeds. The results suggested that the influence of environmental factors on the expression of some QTLs located in different genetic systems should be considered when improving for these amino acids. This study can serve as the foundation for the improvement of these essential amino acids in cottonseeds.

## Introduction

Cotton (*Gossypium hirsutum* L) is a leading fiber crop in the world producing annually both natural fiber and cottonseeds in large quantities. O'Brien et al. (2005) [1] reported that 1.65 kg of cottonseeds could be gained for every 1 kg of lint yielded. Cottonseed kernel is the most nutritious part possessing high oil (28.24–44.05%) and protein (27.83–45.6%) contents [2]. To date, cottonseed oil has been principally used for human consumption, while its protein part for animal feed production. Literature reports the availability of seventeen different kinds of amino acids including essential amino acids like isoleucine, leucine, threonine, methionine, phenylalanine, and lysine in cottonseeds [3], [4]. With the growing world population, the potential for its use as an important source of food is fairly high. For this reason, the improvement on cottonseed quality traits including its amino acid content by genetic breeding is becoming the need of hour. Currently, most of the studies on cottonseeds have been focused on oil and gossypol contents [5], [6] with less emphasis on amino acids. Cherry et al. [7] observed that total amino acid content was mainly controlled by the genotype. Ji et al. [8] analyzed the genetic effects on amino acid content in the seeds of upland cotton and suggested that most amino acid compositions were mainly affected by the dominance effect. Song and Zhang [9] identified QTL for seven amino acids using molecular marker technique, which was carried out only on one genetic system, i.e. embryo genome.

Though cottonseed is a new generation and different from its maternal plant, it still depends on assimilates from the maternal plant during seed development. This suggests that the genetic mechanism of cottonseed quality traits could be directly or indirectly affected by the genetic behavior of maternal plant. That is why the genetic research on cottonseeds is very difficult. Researchers have also reported that cottonseed quality traits are simultaneously controlled by the genetic main effects from different genetic systems including tetraploid embryo and tetraploid maternal plant nuclear genes, as well as their genotype × environment (GE) interaction effects [10]. These reports also suggest that genetic effects related to amino acid content in cottonseeds could be further analyzed based on different genetic systems at the molecular level. Similar studies have been successfully conducted on rice [11], [12]. The analysis of seed quality traits based on the two genetic systems can help further reveal the genetic basis of amino acid content in cottonseeds and serve as the foundation for its quality improvement as a food or feed product.

Amino acids are principally divided into two groups, essential and non-essential. Essential amino acids play a crucial role in the metabolic processes which make the body grow normally but cannot be synthesized in the human body and must be obtained from the foods been eaten [13]. In the present study, investigations to identify QTLs governing essential amino acids except for tryptophan in cottonseeds were conducted using the newly developed QTL mapping method that could analyze embryo and maternal main effects and their QE effects on quantitative traits of seeds in dicotyledonous crops. Seeds of immortal F_2_ populations used in this experiment were obtained from random crosses among a set of 188 RILs. This RIL population which has been successfully used in various genetic studies on cotton [14]–[18] was derived from a hybrid of HS46 and MARKCBUCAG8US-1-88. In this study, the genetic effects including the embryo additive main effect (*a^e^*), embryo dominance main effect (*d^e^*), maternal additive main effect (*a^m^*) and their environmental interactions were analyzed. This work was aimed at revealing in depth the genetic mechanisms of QTLs for essential amino acids, which provide the foundation for the improvement of these essential amino acids in cottonseeds.

## Results

### Phenotypic performance of essential amino acids in cottonseeds

**Figure 1:**
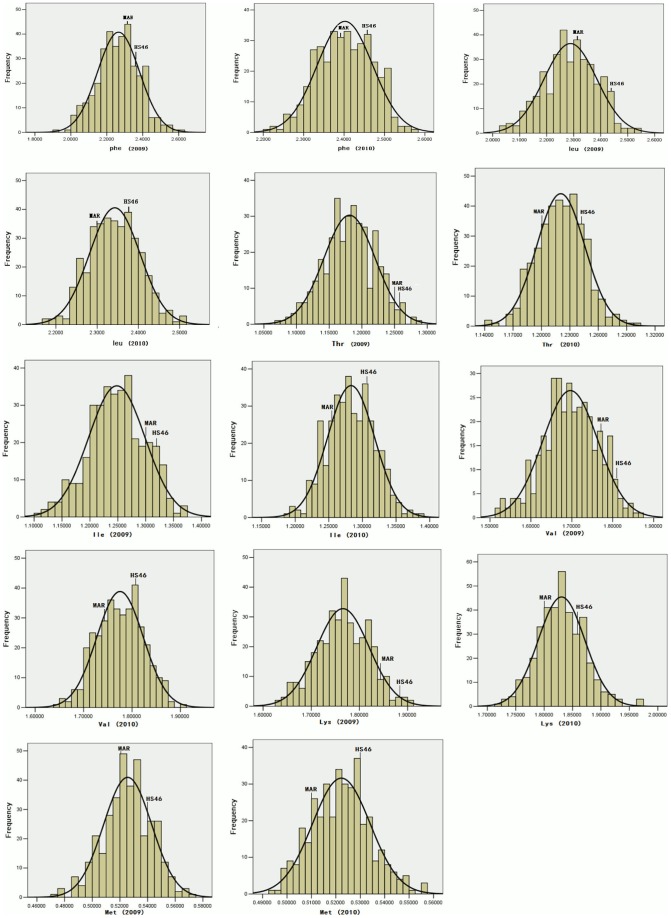
Frequency distribution of essential amino acid contents (%) in IF_2_ population in 2009 and 2010. MAR and HS46 represent mean values of their parents, respectively.

Phenotypic values for essential amino acid contents of the two parents, HS46 and MARKCBUCAG8US-1-88, and the IF_2_ population in 2009 and 2010 are summarized on Table 1. Significant differences between HS46 and MARKCBUCAG8US-1-88 were detected for these traits in two years. The contents of seven essential amino acids in the cottonseeds of HS46 were significantly higher in comparison to those in MARKCBUCAG8US-1-88 in both years. The maximum and minimum values of essential amino acid contents in the IF_2_ population, suggesting a wide variation in essential amino acid contents, were far beyond to the average values of those in HS46 and MARKCBUCAG8US-1-88. This trend implied that there was a transgressive segregation for these quality traits in either direction, which revealed a significant recombination of the QTL for these traits between two parents. The absolute values of skewness and Kurtosis for these seven traits were less than 1, suggesting a normal distribution of these traits within the IF_2_ population, thus making it suitable for QTL analysis. The distributions of their respective phenotypic values in IF_2_ populations are presented in Figure 1. In addition, the average values of six of the seven essential amino acid contents in 2009 were lower than those in 2010, suggesting a certain level of environmental effect on their phenotypic performance.

**Table 1:** The seven essential amino acid contents (%) of parents and the IF_2_ population in 2009 and 2010.

Years	Traits	Mean	IF_2_					Parents	
			S.D	Min	Max	Skew	Kurt	HS46	MAR
	Phe	2.27	0.12	1.93	2.62	−0.01	−0.06	2.33a	2.30b
	Leu	2.29	0.10	2.03	2.53	−0.13	−0.28	2.43A	2.32B
	Lys	1.77	0.05	1.63	1.91	−0.03	−0.24	1.98a	1.97b
2009	Thr	1.18	0.04	1.07	1.29	−0.05	−0.17	1.26a	1.25b
	Ile	1.25	0.05	1.11	1.38	−0.13	−0.28	1.32a	1.30b
	Val	1.70	0.07	1.52	1.86	−0.15	−0.25	1.81a	1.78b
	Met	0.53	0.02	0.47	0.57	−0.20	0.09	0.59a	0.58b
	Phe	2.40	0.07	2.21	2.57	−0.11	−0.51	2.46A	2.37B
	Leu	2.34	0.06	2.18	2.51	0.04	−0.24	2.38A	2.30B
	Lys	1.83	0.04	1.72	1.97	0.26	0.46	1.86A	1.80B
2010	Thr	1.22	0.03	1.14	1.30	0.02	0.17	1.24A	1.20B
	Ile	1.28	0.04	1.19	1.39	0.05	−0.17	1.31A	1.26B
	Val	1.78	0.05	1.64	1.90	−0.08	−0.45	1.81A	1.74B
	Met	0.52	0.01	0.49	0.56	0.18	−0.18	0.53A	0.51B

a, b significance *P* = 0.05; A, B significance *P* = 0.0.

### QTL analysis for essential amino acid contents

**Figure 2:**
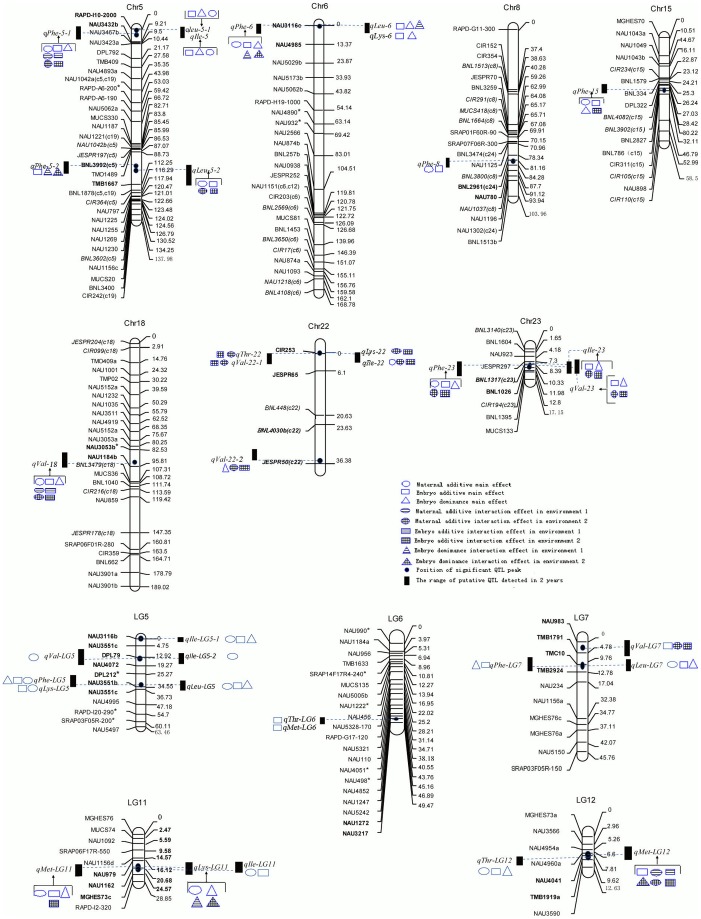
QTL mapping of embryo and maternal plant nuclear genomes for essential amino acid of cottonseeds.

The results of QTL analysis are shown in Tables 2 and 3 as well as in Figure 2. A total of 35 QTLs associated with essential amino acid content were subsequently mapped on chromosomes A5, A6, A8, D15, D18, D22, and D23, as well as on linkage group 5, 6, 7, 11, and 12. Among them, there were thirteen QTLs which explained more than 10% of phenotypic variation. Most QTLs not only had significant genetic main effects from embryo and maternal nuclear genes, but significant QE interaction effects as well. The negative direction of the genetic effects indicated that some alleles from MARKCBUCAG8US-1-88 could increase the contents of these amino acids while a positive QTL effect showed those from HS46 could do the same. The proportion of phenotypic variation attributable to the total genetic main effects and GE interaction effects of QTL were 0.3987 and 0.0136 for leucine, 0.5023 and 0.0379 for phenylalanine, 0.215 and 0.3528 for threonine, 0.2175 and 0.4257 for valine, 0.1037 and 0.2093 for methionine, 0.2232 and 0.3763 for isoleucine, and 0.2237 and 0.3315 for lysine, respectively. These results indicated that the environmental interaction effects were important for the performance of these amino acids.

**Table 2:** Contributions, positions of the QTLs for essential amino acid content in cottonseeds.

Traits	QTL	Chr/LG	Mark interval	Position	Range	R^2^(*a^e^*)	R^2^(*d^e^*)	R^2^(*a^m^*)	R^2^
Leu	*qLeu-5-1*	chr5	RAPD-I10-2000-NAU3432b	1.0	0.0–4.0	0.0169	0.0186	0.0036	0.0391
	*qLeu-5-2*	chr5	BNL3992(c5)-TMB1667	103.1	100.1–117.9	0.1358	0.0000	0.0129	0.1574
	*qLeu-6*	chr6	NAU3116c-NAU4985	0.0	0.0–4.0	0.0129	0.0225	0.0000	0.0403
	*qLeu-LG5*	lg5	DPL212*-NAU3551b	32.3	30.3–35.5	0.0773	0.0030	0.0269	0.1072
	*qLeu-LG7*	lg7	TMC10-TMB2924	9.8	7.8–10.8	0.0635	0.0022	0.0023	0.0680
Phe	*qPhe-5-1*	chr5	RAPD-I10-2000-NAU3432b	3.0	0.0–5.0	0.0336	0.0199	0.0073	0.0693
	*qPhe-5-2*	chr5	BNL3992(c5)-TMB1667	101.1	99.1–104.1	0.1054	0.0000	0.0000	0.1198
	*qPhe-6*	chr6	NAU3116c-NAU4985	0.0	0.0–4.0	0.0222	0.0130	0.0019	0.0394
	*qPhe-8*	chr8	BNL2961(c24)-NAU780	81.2	79.3–84.2	0.0174	0.0000	0.0186	0.0360
	*qPhe-15*	chr15	BNL3902(c15)-BNL2827	28.0	26.2–29.4	0.0354	0.0020	0.0204	0.0590
	*qPhe-23*	chr23	BNL1317(c23)-BNL1026	8.4	6.2–12.0	0.0619	0.0077	0.0066	0.0877
	*qPhe-LG5*	lg5	DPL212*-NAU3551b	32.3	29.3–35.5	0.0793	0.0030	0.0268	0.1091
	*qPhe-LG7*	lg7	TMC10-TMB2924	9.8	6.8–10.8	0.0188	0.0011	0.0000	0.0199
Thr	*qThr-LG6*	lg6	NAU1272-NAU3217	46.9	45.2–47.9	0.0213	0.0000	0.0000	0.0213
	*qThr-22*	chr22	CIR253-JESPR65	0.0	0.0–3.0	0.0000	0.0000	0.0000	0.3528
	*qThr-LG12*	lg12	NAU4041-TMB1919a	8.8	7.8–9.6	0.1361	0.0128	0.0448	0.1937
Val	*qVal-18*	chr18	NAU3053b-NAU1184b	91.5	88.5–96.8	0.0394	0.0080	0.0078	0.0794
	*qVal-22-1*	chr22	CIR253-JESPR65	0.0	0.0–3.0	0.0000	0.0000	0.0000	0.1944
	*qVal-22-2*	chr22	BNL4030b(c22)-JESPR50(c22)	35.6	31.6–35.6	0.0000	0.0056	0.0000	0.0146
	*qVal-23*	chr23	BNL1317(c23)-BNL1026	9.4	7.3–11.3	0.0922	0.0314	0.0000	0.3120
	*qVal-LG5*	lg5	DPL79-NAU4072	14.9	8.8–16.9	0.0000	0.0000	0.0214	0.0214
	*qVal-LG7*	lg7	NAU983-TMB1791	3.0	1.0–5.8	0.0117	0.0000	0.0000	0.0214
Met	*qMet-LG6*	lg6	NAU1272-NAU3217	46.9	46.2–47.9	0.1037	0.0000	0.0000	0.1037
	*qMet-LG11*	lg11	NAU979-NAU1162	20.1	18.1–22.7	0.0101	0.0140	0.1000	0.1354
	*qMet-LG12*	lg12	NAU4041-TMB1919a	7.8	6.6–9.6	0. 0200	0.0000	0.0000	0.2010
Ile	*qIle-5*	chr5	RAPD-I10-2000-NAU3432b	0.0	0.0–5.0	0.0151	0.0214	0.0078	0.0443
	*qIle-LG11*	lg11	NAU1162-MGHES73-c	20.7	18.1–22.7	0.0085	0.0000	0.0551	0.0636
	*qIle-22*	chr22	CIR253-JESPR65	0.0	0.0–3.0	0.0000	0.0000	0.0084	0.2302
	*qIle-23*	chr23	BNL1317(c23)-BNL1026	8.4	6.2–11.3	0.1520	0.0083	0.0000	0.1629
	*qIle-LG5-1*	lg5	NAU3116b-NAU3551c	0.0	0.0–2.0	0.0178	0.0133	0.0384	0.0695
	*qIle-LG5-2*	lg5	DPL79-NAU4072	12.9	9.8–15.9	0.0000	0.0000	0.0139	0.0390
Lys	*qLys-6*	chr6	NAU3116c-NAU4985	0.0	0.0–4.0	0.0098	0.0126	0.0000	0.0224
	*qLys-22*	chr22	CIR253-JESPR65	0.0	0.0–3.0	0.0000	0.0000	0.0000	0.3255
	*qLys-LG5*	lg5	NAU3551b-NAU3551c	34.5	31.3–35.5	0.1261	0.0000	0.0565	0.1826
	*qLys-LG11*	lg11	NAU979-NAU1162	20.1	18.1–22.7	0.0000	0.0120	0.0067	0.0247

R^2^ (*a^e^*), R^2^ (*d^e^*) and R^2^ (*a^m^*), represents the phenotypic variations explained by the *a^e^*, *d^e^* and *a^m^*, respectively.

R^2^, Phenotypic variation explained by a single QTL.

**Table 3:** Genetic main effects and QE interaction effects from the QTLs of embryo and maternal plant for essential amino acid contents in cottonseeds.

Traits	QTL	*a^e^*	*d^e^*	*a^m^*	*a^e^*E_1_	*a^e^*E_2_	*d^e^*E_1_	*d^e^*E_2_	*a^m^*E_1_	*a^m^*E_2_
Leu	*qLeu-5-1*	−0.0091**	−0.0213**	0.0067**	−0.0005	0.0006	−0.0051	0.0050	0.0005	−0.0005
	*qLeu-5-2*	−0.0258**	0.0051	0.0126**	0.0002	0.0067*	−0.0069	0.0067	−0.0002	−0.0064*
	*qLeu-6*	0.0080**	0.0235**	−0.0014	−0.0003	−0.0005	0.0088*	−0.0091	0.0003	0.0005
	*qLeu-LG5*	−0.0195**	−0.0086**	0.0182**	−0.0003	−0.0013	−0.0041	0.0041	0.0003	0.0014
	*qLeu-LG7*	−0.0176**	−0.0074**	0.0053**	0.0005	0.0002	0.0006	−0.0006	−0.0005	−0.0002
Phe	*qPhe-5-1*	−0.0164**	−0.0283**	0.0121**	−0.0065*	0.0073*	−0.0029	0.0029	0.0063*	−0.0072**
	*qPhe-5-2*	−0.0291**	0.0008	0.0054	0.0004	0.0040	−0.0191**	0.0202**	−0.0003	−0.0041
	*qPhe-6*	0.0134**	0.0229**	−0.0061**	−0.0005	−0.0005	0.0079*	−0.0077*	0.0005	0.0006
	*qPhe-8*	0.0118**	0.0027	−0.0193**	0.0004	−0.0001	0.0006	−0.0006	−0.0004	0.0001
	*qPhe-15*	0.0169**	−0.0091**	−0.0203**	0.0005	−0.0043*	−0.0003	0.0004	−0.0005	0.0041
	*qPhe-23*	0.0223**	−0.0176**	−0.0115**	0.0005	−0.0098**	−0.0035	0.0036	−0.0005	0.0094*
	*qPhe-LG5*	−0.0252**	−0.0110**	0.0232**	−0.0002	−0.0009	−0.0006	0.0007	0.0002	0.0009
	*qPhe-LG7*	−0.0123**	−0.0066*	0.0000	0.0003	−0.0006	0.0028	−0.0027	−0.0003	0.0006
Thr	*qThr-LG6*	−0.0035**	0.0025	−0.0017	0.0003	0.0001	0.0019	−0.0019	−0.0003	−0.0001
	*qThr-22*	−0.0014	0.0005	−0.0014	−0.0002	0.0047**	0.0005	−0.0005	0.0002	−0.0046**
	*qThr-LG12*	−0.008**	−0.0067*	0.0047**	0.0000	0.0016	−0.0033	0.0031	0.0000	−0.0016
Val	*qVal-18*	−0.0117**	−0.0118**	0.0058*	−0.005*	0.0079**	−0.0002	0.0002	0.0054*	−0.008**
	*qVal-22-1*	−0.0009	−0.0034	−0.0024	0.0000	0.0064**	0.0006	−0.0006	0.0001	−0.0063**
	*qVal-22-2*	−0.0021	0.0099**	−0.0020	0.0001	0.0058**	−0.0003	0.0003	−0.0001	−0.0054**
	*qVal-23*	0.009**	−0.0075**	−0.0020	0.0030	−0.008**	−0.0002	0.0002	−0.0031	0.0082**
	*qVal-LG5*	0.0011	−0.0014	0.0097**	−0.0040	−0.0004	−0.0001	0.0001	0.0039	0.0004
	*qVal-LG7*	−0.0064**	−0.0053	0.0024	−0.0003	−0.0058**	0.0001	0.0000	0.0002	0.0058**
Met	*qMet-LG6*	−0.0017**	0.0015	−0.0005	0.0003	0.0000	0.0009	−0.0009	−0.0003	0.0000
	*qMet-LG11*	−0.0028**	0.0036**	0.0009*	−0.0001	0.0009*	0.0014	−0.0014	0.0001	−0.0009
	*qMet-LG12*	−0.0017**	−0.0018	0.0007	−0.0021**	0.0023**	−0.0024	0.0025*	0.002**	−0.0023**
Ile	*qIle-5*	−0.0041**	−0.011**	0.0033**	−0.0001	0.0002	−0.0038	0.0038	0.0001	−0.0002
	*qIle-LG11*	0.0031**	0.0028	−0.0088**	0.0007	0.0014	0.0012	−0.0012	−0.0007	−0.0013
	*qIle-22*	0.0014	−0.0022	−0.0043**	0.0001	0.0046**	0.0021	−0.0021	−0.0001	−0.0048**
	*qIle-23*	0.0051**	−0.0057**	−0.0001	0.0003	−0.0041**	−0.0003	0.0003	−0.0003	0.004**
	*qIle-LG5-1*	0.0056**	−0.0108**	−0.0091**	0.0004	0.0000	−0.0003	0.0003	−0.0004	0.0000
	*qIle-LG5-2*	0.0012	0.0002	0.0055**	−0.0019	−0.0014	−0.0002	0.0002	0.0019	0.0014
Lys	*qLys-6*	0.0048**	0.0122**	0.0003	−0.0003	−0.0010	0.0050	−0.0050	0.0003	0.0009
	*qLys-22*	−0.0016	−0.0018	−0.0019	−0.0006	0.0064**	0.0023	−0.0023	0.0006	−0.0066**
	*qLys-LG5*	−0.013**	−0.0007	0.0098**	−0.0004	−0.0008	−0.0019	0.0018	0.0004	0.0007
	*qLys-LG11*	−0.0009	0.0119**	−0.0045**	0.0002	0.0013	0.0069*	−0.0069*	−0.0002	−0.0013

Notes: *a^e^*, embryo additive main effect; *d^e^*, embryo dominance main effect; *a^m^*, Maternal additive main effect; *a^e^*E_1_ and *a^e^*E_2_, embryo additive interaction effects in environment 1 and environment 2; *d^e^*E_1_ and *d^m^*E_2_, embryo dominance interaction effects in environment 1 and environment 2, *a^m^*E_1_ and *a^m^*E_2_ maternal additive interaction effects in environment 1 and environment 2, respectively. *P = 0.05; **P = 0.01.

#### Leucine

Five QTLs for leucine content were detected in two environments with three of them being identified on chromosome A5 and A6. *qLeuC-5-2* was mapped between markers BNL3992(c5) and TMB1667 on chromosome A5, explaining 15.74 % of phenotypic variation. It had the largest *a^e^* and the second largest *a^m^*, and also the largest *a^e^*E and *a^m^*E in 2010. The results indicated that this QTL was very important for improving leucine content. *qLeuC-6* located between markers NAU3116c and NAU4985 on chromosome A6, had a significant embryo genetic main effect. Its *d^e^* was the largest among five QTLs for leucine content, suggesting strong heterosis in this locous. The remaining three QTLs, namely *qLeuC-5-1*, *qLeuC-LG5*, and *qLeuC-LG7* had extremely significant *a^e^* and *a^m^*, and *d^e^*. However, there were no environmental interaction effects found, suggesting that the expression of these QTLs were stable. In addition, the additive effects of all these QTLs accounted for 36.1% of phenotypic variation, which is greater than that of the dominance effects. This showed a major influence from the additive effects in the expression of all QTLs for leucine content.

#### Phenylalanine

A total of eight QTLs for phenylalanine content, explaining 55.8 % of PV, were detected. Six of them were mapped on chromosome A5, A6, A8, A13, D15, and D23, namely *qPheC-5-1*, *qPheC-5-2*, *qPheC-6*, *qPheC-8*, *qPheC-15* and *qPheC-23*. This result showed that the genetic control for phenylalanine content was distributed over several chromosomes. *a^e^* was significant for all eight QTL and most of them had significant *d^e^* and *a^m^*, which indicated the genetic control for this traits across genetic systems. Significant environmental interaction effects were also found for five QTLs, indicating the importance of the environment in the performance of phenylalanine. *qPheC-5-2*, having largest *a^e^*, was identified in the region of 5 cM between two markers BNL3992(c5) and TMB1667. Except for *d^e^*E, neither *d^e^*, *a^m^* nor other environmental interaction effects were detected. These result showed that the QTL expressed only in the embryo genome. In addition, the alleles from MARKCBUCAG8US-1-88 at this locus increased more phenylalanine content than that from HS46, although this parent was at a lower value for the trait. *qPheC-LG5* explaining 10.91% of phenotypic variation was identified in the interval between markers DPL212* and NAU3551b on linkage group 5. It had the largest additive main effects including *a^e^* and *a^m^* but no significant environmental interaction. This showed it was a stable and major QTL. *qPheC-5-1* with the largest embryo dominance main effect, had significant embryo and maternal additive main effects and their environmental interaction, resulting in a very complex expression. The genotype of MARKCBUCAG8US-1-88 was in the direction of increasing phenylalanine content as shown by the *a^e^*, *d^e^* and *a^e^*E1. Regarding to GE interaction effects of QTL, the magnitude and direction of the same type of environmental interaction varied in different environments.

#### Threonine

Three QTLs (*qThrC-22* and *qThrC-LG6*, *qThrC-LG12*) for threonine content were mapped on chromosome D22, and linkage group 6 and 12, respectively. *qThrC-22* had significant embryo and maternal additive environmental interaction in 2010. The allele on this locus from HS46 and MARKCBUCAG8US-1-88 increased 0.47% and 0.46% of threonine content, respectively, by embryo and maternal additive environmental interaction in 2010. This indicated environment 2 was more important to the expression of this QTL. *qThrC-LG12* which contributed to 19.37% of phenotypic variation, had significant genetic main effects including *a^e^*, *d^e^* and *a^m^*. The absence of a significant QTL × environment interaction suggests that this QTL was stable. *qThrC-LG6,* explaining 2.13% of phenotypic variation had only one notable *a^e^*, suggesting the expression of this QTL was very simple and controlled only by the embryo additive main effect.

#### Valine

A total of 6 QTLs for valine content were detected on five linkage groups including three chromosomes. Five QTLs had significant environmental interaction besides some significant genetic main effects, indicating that the environmental factor was important for this trait. *qValC-23*, whose genetic main effect accounted for 12.36% of phenotypic variation, was found to have no significant maternal additive effect, indicating this QTL expressed mainly in the embryo genome. *qValC-LG5* had only maternal additive main effect, the value of which was the largest among the QTLs for valine content. It implied that it could more effectively increase valine content if this locus in the maternal plant was selected. In addition, many significant genetic effects from qValC-18 and qValC-LG7 in the negative direction also suggested that the alleles at these loci from MARKCBUCAG8US-1-88 could increase valine content.

#### Methionine

Three QTLs for methionine content, all with significant embryo additive main effects, were identified on different linkage groups. The embryo additive main effect accounting for large proportion of the phenotypic variation showed that it was very important for increasing methionine content. *qMetC-LG11* had significant embryo dominance main effect and the direction of its embryo additive and maternal effects was opposite. These results revealed that this trait was simultaneously controlled by the embryo and maternal genomes where the expression of this QTL was inconsistent.

#### Isoleucine

Six QTLs for isoleucine contents were distributed on three linkage groups with three of them defined on chromosome A5, D22 and D23, as well as the other three on lingkage group 5 and 11. The two QTLs on linkage group 5 were different with regards to the direction of embryo dominance main effect and maternal additive main effect. Two QTLs (*qIleC-22* and *qIleC-23*) with higher heritability were affected by environmental condition due to large proportion of the phenotypic variation attributable to GE interaction. *qIleC-23* had no significant maternal additive main effects, suggesting this QTL was primarily affected by the embryo genome. *qIleC-5* had significant embryo and maternal additive main effects and embryo dominance main effects. This QTL was stable due to no significant environmental interaction. *qIleC-LG11*, located between NAU979 and NAU1162 on the linkage group 11, had only significant embryo and maternal additive effects with higher values. The maternal additive effect accounted for large proportion of the total genetic effect of this QTL, although its heritability (6.36%) was relatively low. These results indicated it still was important in the improvement of isoleucine content.

#### Lysine

Four QTLs for lysine content on chromosome A6 and D22 as well as linkage group LG5 and LG11 were detected. *qLysC-6* had only significant embryo additive and dominant effect, suggesting these QTLs were controlled by the embryo genome with insignificant environmental influence. Genetic main effects were not found in *qLysC-22* which had significant additive environmental interaction in environment 2, suggesting it could be important in this special environment. *qLysC-LG5* explaining 12.61% and 5.65% of phenotypic variation by embryo and maternal additive main effects, respectively, had only significant embryo and maternal additive main effects, which showed that it could be stable and effective when used for marker-assisted selection. The negative *a^e^* value of this QTL implied that the allele from MARKCBUCAG8US-1-88 in this locus could increase 0.013% of lysine content. For *qLysC-LG11*, maternal additive main effect, embryo dominance main effect and its environmental interaction effect were significant. However, the phenotypic variation attributable to embryo dominance main effect and its environmental interaction of this QTL accounted for large proportion of the total phenotypic variation. It showed that the expression of this QTL was controlled principally by embryo dominant main effect and its environmental interaction. It was suggested furthermore that this QTL could be very unstable when used for increasing the lysine content. Among these four QTL, two of them had high dominance main effect values, indicating a strong heterosis for lysine content at the molecular level.

### Co-localization of QTLs

A number of QTLs for essential amino acid were found to be co-localized at the same positions. For instance, *qPheC-5-1*, *qLeuC-5-1* and *qIleC-5* on chromosome A5, *qPheC-6* and *qLeuC-6* on chromosome A6, *qThrC-22*, *qValC-22-1*, *qLysC-22* and *qIle-22* on chromosome D22 were found at the same positions. Other co-localized QTLs including *qPheC-5-2* and *qLeuC-5-2* on chromosome A5, *qIleC-LG5-1* and *qValC-LG5* on LG5, *qValC –LG7* and *qPheC-LG7* on LG7, may be tightly linked. These results indicated that multiple essential amino acid contents could be simultaneously improved.

## Discussion

Being used as food for human and feed for animals, cottonseeds have been mainly studied for its oil and gossypol contents [6], [22], [23]. Little attention has been paid to its amino acid contents because of high cost involved in measuring these traits. Cottonseeds could become fully edible food by humans if gossypol is eliminated and the different kinds of amino acids that are essential for human health are improved. It is therefore believed that present study will be of practical significance in cottonseed breeding especially for essential amino acid contents.

Many studies have confirmed that quantitative traits can be divided into single Mendelian quantitative trait loci (QTLs) [24],[25]. Most of seed quality traits are complex quantitative traits because the seed genome is different from that of the maternal plant genome. For cottonseeds, the QTLs located on one chromosome may be simultaneously influenced by embryo and maternal genomes [10]. Therefore, the genetic effects of these QTLs can be inferred from these two genomes. In addition, environmental factors are important in the performance of quantitative traits. Thus, the influence of the environment on QTLs associated with seed quality traits should be considered. This will help breeders adopt more effective strategies in seed quality improvement.

Compared to previous studies [26], [27], the genetic information on amino acids in cottonseeds in the present study were more thorough. Phenylalanine and leucine contents were primarily controlled by genetic main effects, which were similar to the results by Cherry [7]. Ji et al.[8], proposed, basing on phenotypic data, that essential amino acid except for methionine were primarily controlled by dominance effect. However, in the present study, the dominance effect of QTLs for these traits only accounted for small proportion of phenotypic variance. Differences between them appeared to be possibly due to their different genetic backgrounds. Song [9] detected three significant QTLs for leucine, phenylalanine and isoleucine on chromosomes D2, A8, and D3, respectively. No QTL for leucine and isoleucine contents was identified on chromosome D2 and D3 in present study. However, four QTLs identified (two for leucine on linkage group 5 and 7 and two for isoleucine on linkage group, 5 and 11) may be related to that on D2 and D3. One QTL for phenylalanine content (*qPheC-8*) mapped on chromosome A8 in the present study may be in the vicinity of that QTL for leucine mapped by Song [9].

In the present study, the results revealed that these seven essential amino acids were simultaneously controlled by genetic main effects and QE interaction effects from the QTLs located in the embryo and maternal nuclear genomes. This is first time to that evidence was provided for that the quality traits in cottonseeds were simultaneously controlled by embryo and maternal genomes [10]. These results were beneficial to the understanding of the molecular genetic mechanism of essential amino acid contents. The application of this information can help breeders adopt more effective strategies for the improvements of these traits, thus insuring better quality is achieved. For example, *qLeuC-5-2*, *qpheC-5-2*, *qMetC-22*, and *qIleC-22*, with notable embryo and maternal additive effect, could be used for marker-assisted selection (MAS). It was also observed that, some QTLs have significant environmental interaction effects beside the significant genetic main effects. Therefore environmental factors should also be considered because environmental interaction effects are varied in different environments. In this experiment, 13 QTLs (*qLeuC-5-2*, *qLeuC-LG5*, *qPheC-5-2*, *qPheC-LG5*, etc) explained more than 10% of phenotypic variation, indicating that they were major QTLs [28]. Some QTLs including *qLeuC-5-1*, *qLeuC-LG7*, *qPheC-8* etc, were also vital because they were stable, although their contributions were relatively small.

In addition, QTLs for different amino acid contents were identified at the same position, which revealed the close relationship between them. It may be caused by the linkage of multiple QTLs or pleiotropic effects of a single gene on multiple traits [29]. This result proved that it is feasible to simultaneously improve the contents of these essential amino acids.

The QTL mapping model and software used in the present experiment have the power to dissect genetic effects of QTLs from different nuclear genomes and to discern different QE interaction effects of the embryo and maternal plant genomes across environments. It helps better understand the genetic mechanisms of seed quality traits at phenotypic and molecular levels. Meanwhile, this QTL mapping model could also be used in the further analysis of genetic effects for other dicotyledonous plants without endosperm.

Near infrared reflectance spectroscopy (NIRS), which is rapid, non-destructive alternative to traditional analytical technique for the prediction of chemical parameters, was used to measure essential amino acid contents in this study [4]. A large number of samples could be rapidly assessed with this technology. However, a calibration equation, which serves as a bridge between spectral data and the prediction of chemical parameters was required based on data obtained by chemical method. In the present study, tryptophan content in cottonseeds was not measured by the chemical method. Thus its equation could not be developed with NIRS making it the only amino acid whose QTL could not be mapped. In addition, the identification of QTLs for methionine content was not as good as other six amino acids due to less accurate calibration equations. However, it still provided some useful information.

## Materials and Methods

### Plant materials

A set of 188 recombinant inbred lines (RILs) and their parents used in this experiment were kindly supplied by USDA-ARS, Starkville, Mississippi, USA in 1999. RILs were developed from an intraspecific hybrid between two upland cotton germplasm, HS46 and MARCABUCAG8US-1-88, with wide genetic differences in yield, fiber quality, disease resistance, and seed quality traits. The F_2_ plants from the intraspecific hybrid were selfed till the F_8_ generation using bulk-base procedure, resulting in the 188 recombinant inbred lines [14]. These materials have been conserved through self-pollination for many years. In this study, every two lines among the 188 RILs were randomly crossed during flowering to produce 376 immortal F_2_ (IF_2_) lines, which were used for QTL analysis.

### Field method

The 188 RILs and their parents (HS46 and MARKCBUCAG8US-1-88) were planted at the experimental farm belonged to Cotton Research Institute, Chinese Academy of Agricultural Sciences, in Sanya, Hainan province, China in 2009 and 2010. The land used for the farm is not privately-owned or protected and it is specially severed for cotton breeders to plant cotton materials in winter. No specific permissions were therefore required to use the land. In addition, cotton is one of the important economic crops in the world. It is cultivated by the farmers near our farm and is not an endangered or protected species. The experiment was laid out in a randomized block design with two replications at 7.0 m length of each plot. The materials mentioned above were grown at a spacing of 0.8 m between rows and 0.25 m between plants. Standard growth practices were performed throughout the growing season. At flowering stage, 376 crosses were randomly made between the 188 RILs according to a diallel mating design. The hybrid seeds produced on an RIL plant formed IF2 population which combined the advantages of the RI population and F2 population. The same crosses were made among the 188 RILs in both years. Seeds of IF2 population and two parents were manually harvested at maturity.

### Sample preparation and trait measurement

Cottonseeds after ginning were delinted and dried. The shells of two hundred seeds from each sample were removed and ground into powder with the Universal High-speed Grinder DFT-50 (Linda Machinery Company Ltd, Wenlin, Zhejiang Province, China). The powdered samples were dried to equilibrium at 25°C with a moisture content of about 7%. The NIR System mode 5000 monochromator (NIR System L Silver spring, MD, USA) was used to scan all samples for spectral information on the essential amino acids [4].

### Linkage map for QTL analysis

A relatively higher density genetic linkage map was constructed in the present study based on the RIL population, using three kinds of molecular markers, SSRs, SRAPs and RAPDs. The genetic map consists of 388 molecular markers mapped on 30 linkage groups. It covers a total length of 1946.22 cM, which accounts for 41.55 % of the whole genome, with an average distance of 5.03 cM between adjacent markers. Out of 30 linkage groups, 15 were identified in 14 chromosomes, with 7 chromosomes in the A sub-genome and the other 7 in the D sub-genome.

### Statistical analysis and QTL mapping

Descriptive statistics including mean, standard deviation, minimum and maximum values, skewness, and kurtosis of the cottonseed essential amino acid contents were calculated using the SPSS 13.0 Data Editor (IBM Corporation, route 100, Somers, NY 10589, USA). A new QTL mapping model developed specifically for the mapping population consisting of an IF_2_ population and two advanced backcross populations was used in this study. The genetic main effects of QTLs in the model included embryo additive main effect (*a^e^*) and embryo dominance main effect (*d^e^*) from tetraploid embryo nuclear genes, and maternal additive main effect (*a^m^*) from tetraploid maternal plant nuclear genes. The model also analyzed the interaction effects of QTL × environment including embryo additive environmental interaction effects (*ae*E), embryo dominance environmental interaction effects (*d^e^*E), and maternal additive environmental interaction effects (*a^m^*E). QTL detection was conducted using QTLNetwork-CL-2.0-Seed which is newly developed software specifically for mapping QTL of embryo traits. The procedure of mixed linear model-based interval mapping was conducted according to the strategy proposed by Yang et al. [19]. An LOD value of 3 was chosen as the threshold to declare a putative QTL. The window size was set at 10 cM and the walking speed was at 1 cM. LOD threshold values were estimated by 1,000 permutations to declare a significant QTL [20]. The Monte Carlo Markov Chain (MCMC) algorithm was used to estimate QTL effects, as well as their QTL × environment interaction effects, and corresponding *P* values. QTL nomenclature was based on Mc Couch et al. [21]. The designation begins with “q”, followed by an abbreviation of the trait name, the location of a QTL on a chromosome or linkage group, and finally, the number assigned to the QTL related trait on a specific chromosome or linkage group. In addition, if a QTL has been identified on a linkage group, then “LG” was placed before the number representing the location of that particular linkage group. If there is only one QTL for a trait on the specific chromosome or linkage group, the last number was omitted in QTL nomenclature.
